# Firing Properties of Genetically Identified Dorsal Raphe Serotonergic Neurons in Brain Slices

**DOI:** 10.3389/fncel.2016.00195

**Published:** 2016-08-03

**Authors:** Boris Mlinar, Alberto Montalbano, Lukasz Piszczek, Cornelius Gross, Renato Corradetti

**Affiliations:** ^1^Department of Neuroscience, Psychology, Drug Research and Children’s Health, University of FlorenceFlorence, Italy; ^2^Mouse Biology Unit, European Molecular Biology LaboratoryMonterotondo, Italy

**Keywords:** serotonergic neurons, neuronal population, pacemaker neurons, firing regularity, oscillatory firing

## Abstract

Tonic spiking of serotonergic neurons establishes serotonin levels in the brain. Since the first observations, slow regular spiking has been considered as a defining feature of serotonergic neurons. Recent studies, however, have revealed the heterogeneity of serotonergic neurons at multiple levels, comprising their electrophysiological properties, suggesting the existence of functionally distinct cellular subpopulations. In order to examine in an unbiased manner whether serotonergic neurons of the dorsal raphe nucleus (DRN) are heterogeneous, we used a non-invasive loose-seal cell-attached method to record α1 adrenergic receptor-stimulated spiking of a large sample of neurons in brain slices obtained from transgenic mice lines that express fluorescent marker proteins under the control of serotonergic system-specific *Tph2* and *Pet-1* promoters. We found wide homogeneous distribution of firing rates, well fitted by a single Gaussian function (*r*^2^ = 0.93) and independent of anatomical location (*P* = 0.45), suggesting that in terms of intrinsic firing properties, serotonergic neurons in the DRN represent a single cellular population. Characterization of the population in terms of spiking regularity was hindered by its dependence on the firing rate. For instance, the coefficient of variation of the interspike intervals (ISI), a common measure of spiking irregularity, is of limited usefulness since it correlates negatively with the firing rate (*r* = −0.33, *P* < 0.0001). Nevertheless, the majority of neurons exhibited regular, pacemaker-like activity, with coefficient of variance of the ISI lower than 0.5 in ~97% of cases. Unexpectedly, a small percentage of neurons (~1%) exhibited a particular spiking pattern, characterized by low frequency (~0.02–0.1 Hz) oscillations in the firing rate. Transitions between regular and oscillatory firing were observed, suggesting that the oscillatory firing is an alternative firing pattern of serotonergic neurons.

## Introduction

In mammals, the dorsal raphe nucleus (DRN) contains the largest population of serotonergic neurons, estimated to be ~9000 in the mouse (Daszuta and Portalier, 1985; Ishimura et al., 1988), 11,500–15,000 in the rat (Descarries et al., 1982; Vertes and Crane, 1997) and ~165,000 in humans (Baker et al., 1991). Early electrophysiological experiments carried out in brain slices and in anesthetized animals have revealed that serotonergic neurons in DRN discharge with a slow (1–2 Hz), regular (clock-like) pattern, suggesting a homogeneous population of pacemaker neurons (Aghajanian et al., 1968; Mosko and Jacobs, 1974, 1976; Aghajanian and Vandermaelen, 1982). Studies in behaving animals further revealed that the firing rate of putative DRN serotonergic neurons is strongly linked to the sleep-wake cycle, showing strong positive correlation with the level of behavioral arousal (McGinty and Harper, 1976; Trulson and Jacobs, 1979; Jacobs and Fornal, 1991). In spite of uncertainty about neuron type identification, data drawn from a larger sample of putative DRN serotonergic neurons in awake animals revealed their heterogeneity with respect to the sleep-wake cycle and suggested the existence of atypical serotonergic neurons, exhibiting spiking activity different from the canonic clock-like pattern (Sakai and Crochet, 2001; Urbain et al., 2006; Sakai, 2011). In recordings from anesthetized animals, a subset of serotonergic neurons was found to discharge with a particular burst-like repetitive mode, characterized by doublets, or occasionally triplets, of closely separated spikes per cycle (Hajós et al., 1995, 1996; Hajós and Sharp, 1996).

By using a juxtacellular labeling method (Pinault, 1996), which greatly improved neuron type identification, it was confirmed that most DRN serotonergic neurons exhibit slow and regular spiking (Allers and Sharp, 2003) and that a subset discharges in burst-like repetitive mode (Hajós et al., 2007). Further studies using juxtacellular labeling revealed both a subset of fast-firing (>8 Hz) serotonergic neurons (Kocsis et al., 2006) and functional differences between single spike and burst firing serotonergic neurons (Schweimer and Ungless, 2010; Schweimer et al., 2011). Recent studies using optogenetic identification of serotonergic neurons have further strengthened the case for the heterogeneity of serotonergic neurons, as atypical, non-clock-like firing neurons have been observed (Cohen et al., 2015). Furthermore, different basal firing rates and reward-related tonic and phasic firing patterns have been reported (Liu et al., 2014; Li et al., 2016). The heterogeneity of DRN serotonergic neurons in behaving animal is at least in part consequential to differences in afferent connections (Warden et al., 2012; Weissbourd et al., 2014), but it could also be due to differences in intrinsic properties of serotonergic neurons. The results of some whole-cell patch clamp studies support this possibility, suggesting diverse subtypes of serotonergic neurons in the DRN (Lowry et al., 2000; Crawford et al., 2010; Calizo et al., 2011; Fernandez et al., 2016). However, evidence of intrinsically heterogeneous classes of serotonergic neurons is far from clear and the possibility that the diversity of serotonergic neurons represents only normal population variability of serotonergic neurons has been raised (Andrade and Haj-Dahmane, 2013).

In order to examine in an unbiased manner whether DRN serotonergic neurons are intrinsically heterogeneous we recorded the spiking activity in a large number of genetically identified serotonergic neurons by using a non-invasive loose-seal cell-attached method. Our data suggest that in terms of their intrinsic spiking properties, serotonergic neurons in the DRN can be considered as a single cellular population, characterized by a wide homogeneous distribution of firing rates and the regularity of spiking proportional to the rate.

## Materials and Methods

### Transgenic Mice

**Figure 1:**
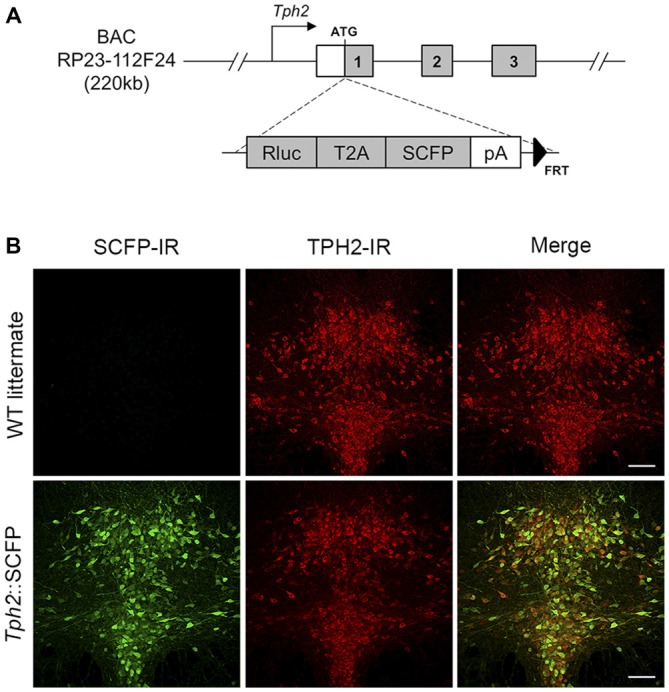
**Generation and characterization of** *Tph2***::SCFP (TSC) transgenic mice. (A)** Scheme of BAC construct used for generation of the *TSC* transgenic mouse line. The Rluc-T2A-SCFP cassette was inserted at the ATG site of the *Tph2* gene in an RP23-112F24 mouse BAC. The modified construct was used for creation of a transgenic mouse line, allowing super cyan fluorescent protein (SCFP) and *Renilla* luciferase expression under *Tph2* promoter. **(B)** Confocal images of SCFP and TPH2 immunoreactivity in 70 μm coronal sections of the dorsal raphe nucleus (DRN) in wild type littermates (upper panel) and *TSC* transgenic mice (lower panel). SCFP expression was detected in the DRN of *TSC* mice with an anti-GFP/CFP antibody (shown in green; left). No signal was seen in wild type littermates. TPH2 expression was detected with an anti-TPH2 antibody (red; middle). In *TSC* mice, the SCFP signal co-localizes with virtually all TPH2-positive neurons in the DRN (yellow in the sum of both channels; right). IR, immunoreactivity. Scale bar: 100 μm.

All animal manipulations were performed according to the European Community guidelines for animal care (DL 116/92, application of the European Communities Council Directive 86/609/EEC) and were approved by the Committee for Animal Care and Experimental Use of the University of Florence. Animals were housed in groups of 3–5 per cage and maintained under standard laboratory conditions (food and water *ad libitum*, 12–12 h light-dark cycle with lights on from 08:00 to 20:00 h, ambient temperature 22 ± 1°C, relative humidity 40–50%). Three transgenic mouse lines with serotonergic system-specific fluorescent protein expression were used. The *Tph2*::SCFP (TSC) transgenic mouse line was produced by pronuclear injection in FVBxFVB embryos of a circular mouse BAC (RP23-112F24, Chori-BACPAC Resources, Oakland, CA, USA) containing 220 kb of the *Tph2* gene in which the Renilla luciferase (Rluc; psiCHECK^TM^, Promega, Fitchburg, WI, USA), in-frame, with the T2A sequence (Holst et al., 2006), followed by super cyan fluorescent protein 3A (SCFP3A) coding sequence (from pSCFP3A-C1; Kremers et al., 2006), bovine growth hormone polyadenylation sequence and an FRT-flanked kanamycin resistance marker (FLP deleted in bacteria before DNA injection) had been inserted at the start codon of the *Tph2* gene (Figure 1A). Founders carrying the transgene were identified and genotyped by PCR. The *TSC* line had stable, high-level transgene expression as measured by Rluc expression (data not shown). Nearly all serotonergic neurons in the DRN were found to express SCFP (Figure 1B). Pet1-Cre::CAG.eGFP (PCG) line (Montalbano et al., 2015) was obtained by crossing Pet1-Cre mice, expressing Cre recombinase in 5-HT neurons by the *Pet1* promoter and enhancer (Dai et al., 2008) with CAG.eGFP reporter mice, carrying an inducible eGFP cassette (Nakamura et al., 2006). Pet1-Cre::Rosa26.YFP (PRY) was obtained by crossing Pet1-Cre mice with ROSA26-stop-YFP reporter mice (Srinivas et al., 2001). All lines were maintained in a pure C57BL/6 strain. Pet1-Cre and CAG.eGFP mice were kindly provided by Prof. K.P. Lesch (University of Würzburg, Würzburg, Germany). ROSA26-stop-YFP reporter mice were purchased from the Jackson Laboratory (Bar Harbor, ME, USA).

### Immunofluorescence

Mice were anesthetized intraperitoneally with Avertin (Sigma-Aldrich, Milan, Italy) and perfused transcardially with 4% paraformaldehyde. Brains were post-fixed overnight at 4°C and sectioned into 70 μm thick slices with a vibratome (Leica Microsystems, Wetzlar, Germany). Free floating sections were stained with primary antibodies overnight at 4°C (1:400 mouse α-TPH, Sigma-Aldrich; 1:800 chicken α-GFP/CFP, Aves Labs, Tigard, OR, USA) and incubated with secondary antibodies for 2 h at room temperature (IgG A488 or IgG A594, Molecular Probes/Thermo Fisher Scientific, Waltham, MA, USA). Confocal microscopy was performed with a TCS-SP5 Laser Scanning System (Leica Microsystems). The images were processed and analyzed using the ImageJ software (ImageJ, National Institutes of Health, Bethesda, MD, USA^1^).

### Loose-Seal Cell-Attached Recordings

**Figure 2:**
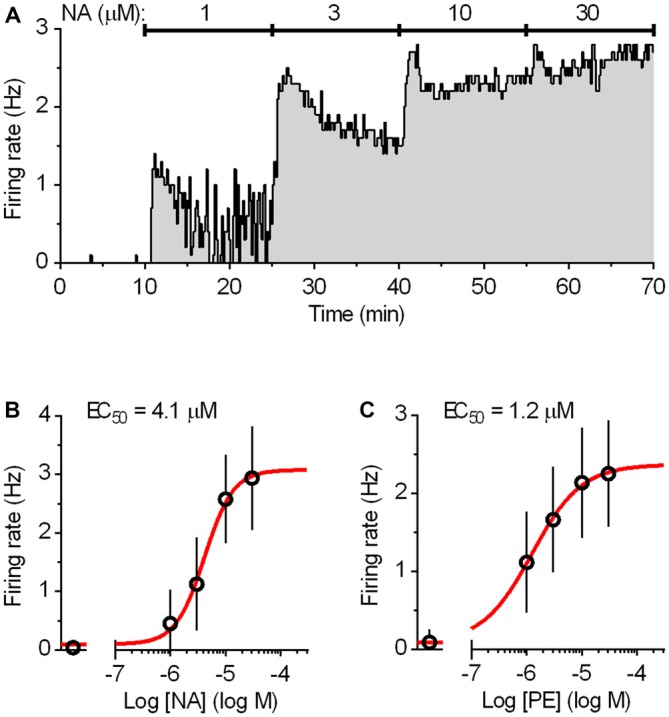
**Dose-response curves for the activation of the DRN serotonergic neurons by noradrenaline (NA) and phenylephrine (PE). (A)** Integrated firing rate histogram (10 s bins) showing the effect of bath application of NA on the firing rate of a DRN serotonergic neuron. Increasing concentrations of NA (15 min each) were applied during the times indicated by solid lines. The firing rate during the last 3 min at each concentration was used for construction of the dose-response curve. **(B)** Average dose-response curve for NA. Symbols represent the mean of eight experiments. Error bars represent SD. Curve (red) represents the best least squares fit to four-parameter logistic equation. **(C)** Average dose-response curve for α1-adrenergic receptor agonist PE constructed in the same way as that of NA. Symbols represent the mean of nine experiments. Error bars represent SD. Curve (red) represents the best least squares fit to four-parameter logistic equation.

Mice (4–28 weeks of age) were anesthetized with isofluorane and decapitated. The brains were rapidly removed and dissected in ice-cold gassed (95% O_2_ and 5% CO_2_) ACSF composed of: 124 mM NaCl, 2.75 mM KCl, 1.25 mM NaH_2_PO_4_, 1.3 mM MgCl_2_, 2 mM CaCl_2_, 26 mM NaHCO_3_, 11 mM D-glucose. The brainstem was sliced coronally into 200 μm thick slices with a vibratome (DSK, T1000, Dosaka, Japan). Slices were allowed to recover for at least 1 h at room temperature and then were individually transferred to a submersion type recording chamber and continuously superfused at a flow rate of 2 ml min^−1^ with oxygenated ACSF warmed to 37°C by a feedback-controlled in-line heater (TC-324B/SF-28, Warner Instruments, Hamden, CT, USA). Slices were allowed to equilibrate for 10–20 min before the beginning of the recording. To reproduce noradrenergic drive that facilitates serotonergic neuron firing during wakefulness (Baraban and Aghajanian, 1980; Levine and Jacobs, 1992), ACSF was supplemented with the natural agonist noradrenaline (NA; 30 μM) or with the α1 adrenergic receptor agonist phenylephrine (PE; 10 μM; Vandermaelen and Aghajanian, 1983). NA and PE were used at minimal concentrations sufficient to produce a full effect on firing (approximately 10 times higher than the respective EC_50_ values; Figure 2). An antioxidant, disodium metabisulfite (Na_2_S_2_O_5,_ 30 μM) was added to NA-supplemented ACSF to prevent NA oxidation. Recordings were done without the addition of synaptic blockers as we had previously established that under identical recording conditions, spiking of serotonergic neurons is not influenced by the antagonist application (Mlinar et al., 2015). Similarly, no 5-HT1A receptor antagonist was applied since autoinhibition by endogenous 5-HT is insignificant under the conditions used (i.e., without supplementing ACSF with the 5-HT precursor Trp; Mlinar et al., 2005). Typically, recordings were done on four slices per animal, and 5–27 neurons were recorded per slice.

Neurons within DRN were visualized by infrared Dodt gradient contrast video microscopy, using a 40× water-immersion objective (N-Achroplan, numerical aperture 0.75, Zeiss, Göttingen, Germany) and a digital CCD camera (ORCA-ER C4742-80-12AG; Hamamatsu, Hamamatsu City, Japan) mounted on an upright microscope (Axio Examiner Z1; Zeiss) controlled by Axiovision software (Zeiss). Loose-seal cell-attached recordings were made from fluorescent protein-expressing neurons, visually identified by using Zeiss FilterSet 46 (eGFP and YFP, excitation BP 500/20, emission BP 535/30) or Zeiss FilterSet 47 (CFP, excitation BP 436/20, emission BP 480/40). Fluorescence was excited using a metal halide lamp (Zeiss HXP 120). Patch electrodes (3–6 MΩ) were pulled from thick-walled borosilicate capillaries (1.50 mm outer diameter, 0.86 mm inner diameter; Corning) on a P-97 Brown-Flaming puller (Sutter Instruments, Novato, CA, USA) and filled with solution containing (in mM): 125 NaCl, 10 HEPES, 2.75 KCl, 2 CaCl_2_ and 1.3 MgCl_2_, pH 7.4 with NaOH. Each pipette was used for several recordings (typically 5–10) and was replaced if tissue debris attached to the tip. When a new patch electrode was used, before touching the cell membrane, the positive pressure was released for several seconds to expose the pipette tip to slice tissue and thus prevent the development of giga-seal. After positioning the pipette in gentle contact with the cell membrane, development of loose seal was monitored by using a voltage-clamp protocol with holding potential of 0 mV and test pulse of 1 mV/100 ms, repeated every second. Weak positive pressure was released and gentle suction was slowly applied until detected spikes increased to 50–100 pA peak-to-peak amplitude. Corresponding seal resistance was in the 10–20 MΩ range. Following the sealing procedure, which lasted 10–30 s, the amplifier was switched to the track (slow voltage clamp) mode and spiking activity was continuously recorded for 2–3 min. Exceptionally, if the firing rate was very low or the spiking pattern appeared anomalous, recordings were prolonged for several minutes and/or the recorded neuron was repatched to confirm the observation. In a minority or recordings the loose seal was established at the beginning of the continuous recording in track mode and the seal resistance was verified at the end of the recording.

Recordings were made using an Axopatch 200B amplifier (Molecular Devices, Sunnyvale, CA, USA) controlled by Clampex 9.2 software (Molecular Devices). Signals were low-pass filtered with a cut-off frequency of 5 kHz (Bessel) and digitized with a sampling rate of 40 kHz (Digidata 1322A, Molecular Devices). After the recording, images of recorded neuron were acquired to document the expression of the fluorescent marker in the recorded neuron, as well as its anatomical location, size and shape.

### Measures for Improving the Reliability of Loose-Seal Cell-Attached Recordings

**Figure 3:**
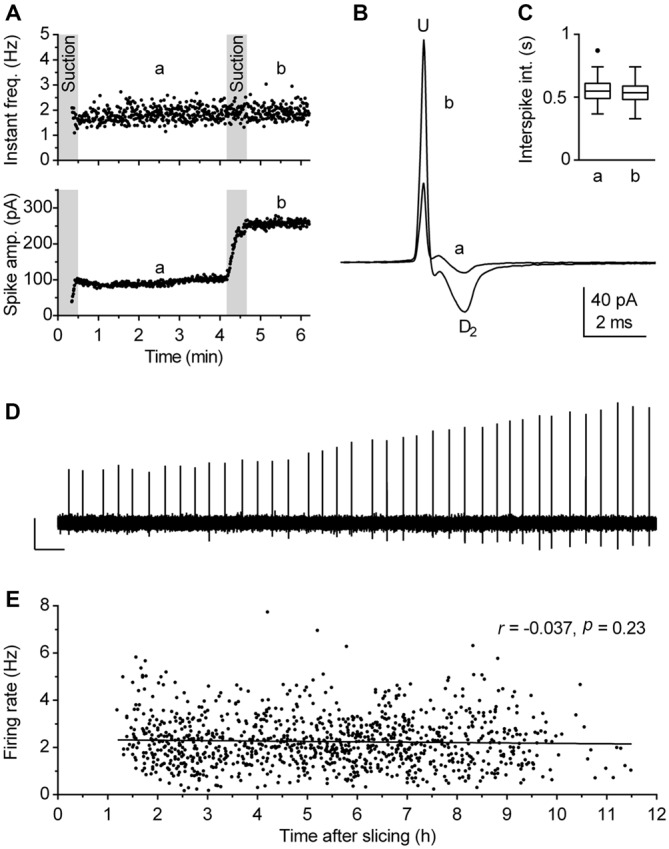
**Validity of firing rate measurement by use of loose-seal cell-attached recordings. (A–D)** The procedure used to verify the lack of the pipette interference with the measurement. **(A)** Time-course of a recording illustrating the procedure. During the first 30 s, gentle suction was applied to the pipette to establish loose-seal cell-attached recording configuration. The pressure was then released and after 30 s, left to allow relaxation of the patched cell membrane, a 3-min-long segment (denoted a, from 1 to 4 min) was acquired for the measurement. Afterward, additional suction was slowly applied until the spike amplitude approximately doubled (lower panel) and the recording was prolonged for an additional 1.5 min (denoted b). Since the firing rate remained essentially the same after the test (second suction), the recording was considered reliable, i.e., free of pipette interference. **(B)** Superimposed average spikes of the same experiment. Spike duration, measured as the interval from the upstroke peak (U) to the second downstroke peak (D_2_), was unchanged by the additional suction. **(C)** Box plot of the same experiments shows no changes in distribution of interspike intervals (ISI). Boxes represent median and the interquartile range (IQR). Whiskers denote 1.5 IQR. **(D)** Trace shows a segment (4:10–4:30 min) of the original recording during which additional (test) suction was applied. Scale bars: 50 pA, 1 s. **(E)** *Post hoc* analysis performed to verify that firing rate of recorded neurons was not influenced by the time passed between slicing and recording. Symbols represent individual neurons. Pearson correlation revealed no correlation between the time after slicing and the firing rate (*r* = −0.037, 95% CI −0.096 to 0.023, *r*^2^ = 0.0014, *P* = 0.23). Lines represent linear regression (Slope −0.017 Hz h^−1^).

Although loose-seal cell-attached recording under visual guidance is a conceptually simple procedure, reliable measurements of spiking activity rely on two critical factors: (I) the absence of interference of the patch pipette with the recorded neuron; and (II) the reliability that recordings are done on healthy cells. The interference of the pipette with the cell membrane, in particular mechanical stress, may compromise estimation of the neuronal firing rate by using cell-attached recordings (see Alcami et al., 2012 for a critical analysis). As we wanted to obtain reliable, artifact-free recordings from a large number of neurons, additional precautions were made in addition to the above described careful sealing procedure. Thus, the segment of the recording obtained during, and in some cases for up to 1 min following, the sealing procedure was not considered for analysis, because, in spite of our careful approach, spiking activity was transiently influenced by sealing in ~30% of cases. In case of doubt that the mechanical stretch (exerted by the pipette pressing the plasma membrane and/or the applied suction stretching it) may have interfered with spiking activity, measurement reliability was verified at the end of the recording by application of an additional pulse of suction to the pipette (Figures 3A–D). In addition, recordings were interrupted and data discarded if the baseline current (i.e., segments between spikes), monitored online, showed any sign of instability, such as variable amplitude, irregular shape, inward current events, likely to be caused by opening of stretch-activated channels (Suchyna et al., 2009; Alcami et al., 2012).

Viability of preparation is the second crucial factor which may influence results obtained in brain slice recordings. We have previously observed that in unhealthy or aging slices serotonergic neurons fire at a lower rate and ultimately become silent. Under the experimental conditions used in this study, slices were typically viable for 8–10 h. Because our principal objective was to define firing characteristics of a population of neurons, we used several online and *post hoc* criteria to ensure that recordings were done from healthy neurons. First, the neurons selected for recordings were 30–60 μm distant from the slice surface, had an overall healthy appearance and clearly visible intact primary neurites. Second, no further recordings were done in slices in which fluctuations in baseline current or the presence of afterspike (tail) currents were detected more than once. We found that the appearance of afterspike current represents a particularly reliable symptom of decreased viability of brain slices, as it is absent in technically valid recordings from healthy neurons, but often gradually develops during recordings in aging slices. In extreme cases, spike shape can change from its normal form corresponding to the time derivative of action potential to an action potential-like shape, characterized by wider spike and the afterspike current with the time course corresponding to the after-hyperpolarization. We believe that such a deformation of spike shape reflects a decreased resistance of the membrane patch caused by compromised integrity of cell membrane in aging or unhealthy neurons. Third, the reliability of recordings was additionally verified during analysis and several experiments in which there was an increase in spike width and/or the appearance of interspike current were excluded from further analysis. Finally, as a precaution, the order of recordings was scrambled on different days, both regarding slices in respect to their rostrocaudal position as well as regarding location of neurons in a given slice. The validity of these criteria were confirmed *post hoc* by lack of correlation between the firing rate of neurons accepted for analysis and the time interval between slicing and recording for each experimental day (not shown) as well as for the pooled data (Figure 3E).

### Anatomical Location of Recorded Neurons

Location of recorded neurons in the slices were documented immediately after recording. In addition, for each slice, brightfield images (5× objective) and fluorescence images or stacks (10× objectives) were acquired and then stitched offline using ImageJ software to obtain composite images of an entire slice. For each animal, the rostrocaudal level of slices (distance from bregma) was first assigned based on comparisons with a mouse stereotaxic atlas (Paxinos and Franklin, 2001) and then used to reconstruct the stereotaxic coordinates of recorded neurons. The expected precision of the coordinates is ≤10 μm for the lateral axis and ≤50 μm for rostrocaudal and dorsoventral axes. Subdivisions of DRN are based on the observed distribution pattern of serotonergic neurons and the atlas (Paxinos and Franklin, 2001). Rostrocaudal divisions follow suggestions by Abrams et al. (2004).

### Analysis

Spike detection was performed using the event detection routine of Clampfit 9.2 software. Spikes were inspected by eye to assure that there are no false or missed events. Spike duration (width) was determined from the shape of averaged spikes by measuring the interval between the spike upstroke and the second downstroke (see Figure 3B). It was determined only for spikes that had a well-defined second downstroke peak (D_2_; ~90% of neurons). The somatic surface area of recorded neurons was measured using the ImageJ freehand tracing tool. To characterize spiking characteristics, the following parameters were calculated for each recorded neuron: firing rate (number of spikes over time interval); SD of instantaneous frequency; COV of instantaneous frequency (SD of instantaneous frequency/mean instantaneous frequency); SD of interspike intervals (ISI); and COV of ISI (SD of ISI/mean ISI). Parametric tests were used for statistical analysis, i.e., ANOVA test with Tukey’s multiple comparison *post hoc* test and unpaired *t*-test. Pearson’s test and multivariate multiple regression were used to assess for correlation between variables. When appropriate, results of non-parametric tests were reported in addition to those of parametric tests. Data are reported as mean ± SD and median ± interquartile range (IQR). Statistical analysis was performed using Prism 6 software (GraphPad Software, San Diego, CA, USA) with the exception of multivariate multiple regression, which was done using STATA version 14 software (StataCorp LP, College Station, TX, USA).

## Results

### Firing Rate

**Figure 4:**
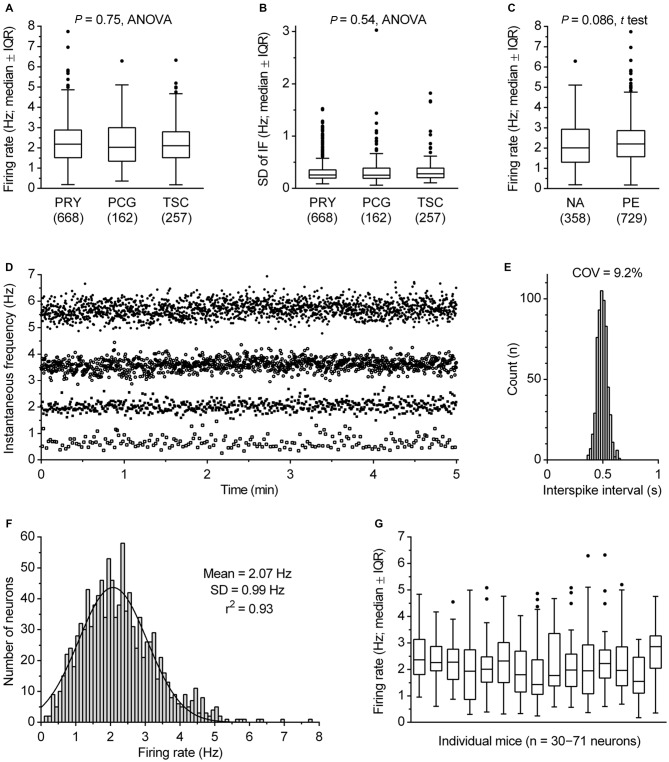
**Firing rate of serotonergic neuron population. (A)** Comparison of firing rates in transgenic mice lines used in this study. Boxes represent medians and the IQR. Whiskers denote 1.5 IQR. PET1-CRE::ROSA26.YFP (PRY), PET1-CRE::CAG.EGFP (PCG) and TSC stay for PRY, PCG and Tph2-SCFP mouse line, respectively. The number of recorded neurons is indicated in parenthesis. **(B)** Comparison of SD of instantaneous firing (IF) in three mouse lines. **(C)** Comparison of firing rates obtained in the presence of 30 μM NA and 10 μM PE. Boxes represent medians and the IQR. Whiskers denote 1.5 IQR. **(D)** Superimposed time-course of four representative recordings covering firing rate range typical for DRN serotonergic neurons. In the examples shown, firing rates were 5.69, 3.59, 2.02 and 0.62 Hz, while SD of instantaneous frequency were 0.29, 0.22, 0.19 and 0.22 Hz, respectively. **(E)** ISI histogram of 2.02 Hz-firing neuron shown in **(C)**. COV denotes variation coefficients of ISI. **(F)** Firing rate histogram of DRN serotonergic neuron population. Curve represents best fit by single Gaussian function. **(G)** Box plot illustrating variability in firing rate of serotonergic neurons in individual animals. Boxes represent medians and the IQR. Whiskers denote 1.5 IQR.

The α1 adrenergic receptor-driven spiking activity of fluorescent protein-marked DRN serotonergic neurons was examined in three transgenic mouse lines to reduce the likelihood of peculiarities potentially caused by genetic modifications. In the PRY and PCG lines, serotonergic system-specific expression of YFP and eGFP, was achieved by Pet-1 promoter (Hendricks et al., 1999; Pfaar et al., 2002), while in the *TSC* line serotonergic system-specific expression of SCFP was achieved by Tph2 promoter. A comparison of data obtained by using all three lines showed that there were no significant differences across the lines in firing rate (*F*_(2,1084)_ = 0.29, *P* = 0.75, ANOVA; range: mean, 2.21–2.27 Hz; median, 2.03–2.18 Hz; Figure 4A), regularity of firing (SD of instantaneous frequency: *F*_(2,1084)_ = 0.62, *P* = 0.54, ANOVA; *P* = 0.22, Kruskal-Wallis; Figure 4B) and firing pattern (see below). Therefore, data obtained from all three lines will be considered as a uniform group. The activity of serotonergic neurons was examined in two conditions designed to fully facilitate their firing, i.e., in the presence of 30 μM NA (NA) and in the presence of 10 μM of the α1 receptor agonist PE (PE). As shown in Figure 4C, similar findings were found under both conditions. Serotonergic neurons exhibited an average firing rate of 2.17 ± 1.13 Hz in NA (mean ± SD, range 0.19–6.29 Hz, median = 2.01 Hz, *n* = 358) and 2.29 ± 1.02 Hz in PE (mean ± SD, range 0.18–7.74 Hz, median = 2.20 Hz, *n* = 729). Although the firing rate of serotonergic neurons was on average slightly lower in NA than in PE, the difference was non-significant (*P* = 0.086, *t* test with Welch’s correction) and all subsequent analysis was done on pooled data.

The great majority of serotonergic neurons exhibited steady spiking, with the firing rate ranging from 0.30 to 5.81 Hz in 99% of recorded neurons (e.g., Figures 4D,E). In pooled data from all recordings, serotonergic neurons exhibited an average rate of 2.25 ± 1.06 Hz (mean ± SD, range 0.18–7.74 Hz, COV = 46.9%, median = 2.13 Hz, *n* = 1087). The frequency distribution of firing rates was well fit by a single Gaussian function (mean = 2.07 Hz, 95% CI 2.00–2.14 Hz; SD = 0.99 Hz, 95% CI 0.92–1.06 Hz; *r*^2^ = 0.93, Figure 4F), whereas fitting with a sum of two Gaussians was ambiguous, suggesting that in terms of firing rate DRN serotonergic neurons represent a homogeneous population with a wide distribution of firing rates. Variability in firing rate of serotonergic neurons was evident in recordings from individual mice. For experimental days in which recordings were done from at least thirty serotonergic neurons from a single animal, the mean firing rate ranged from 1.75 to 2.73 Hz, with SD in 0.70–1.30 Hz range and COV in 29.2–63.3% range (Figure 4G). Similar findings were observed in individual slices and in sequences of recording from neighboring neurons (not shown). Additional *post hoc* analysis showed no significant difference in firing rate of serotonergic neurons with respect to animal age (range 27–195 days; *r* = 0.040, *P* = 0.18, Pearson) or sex (*P* = 0.26, *t* test; males, 2.24 ± 1.06 Hz, mean ± SD, *n* = 843; females, 2.32 ± 1.04 Hz, mean ± SD, *n* = 244).

**Figure 5:**
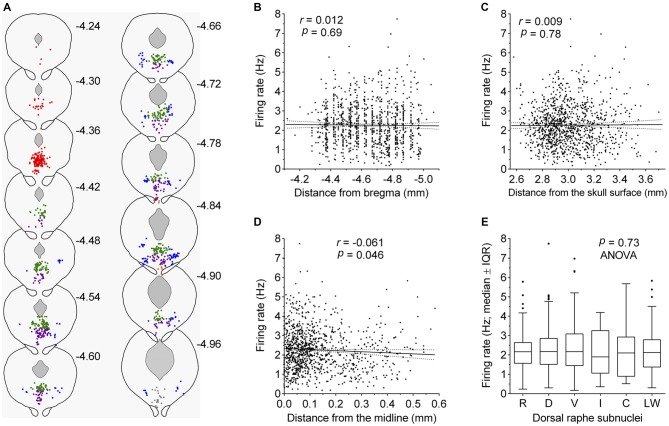
**Firing rate of serotonergic neuron is constant across the DRN. (A)** Coronal sections showing the anatomical location of recorded serotonergic neurons; adaptations from Paxinos and Franklin (2001). Numbers indicate distance of the section from bregma. Symbols represent individual neurons. Colors indicate dorsal raphe subnuclei: red, rostral; green, dorsal; violet, ventral; orange, interfascicular; gray, caudal; blue, lateral wings. **(B–D)** Correlation between the firing rate and neuron location along the rostrocaudal axis **(B)**, the dorsoventral axis **(C)**, and the lateral distance from the midline **(D)**. In **(C)**, numbers on abscissa correspond to numbers on the right margin of coronal plates in mouse brain atlas (Paxinos and Franklin, 2001) and represent dorsoventral distance from the horizontal plane passing through bregma and lambda on the surface of the skull. Symbols represent individual neurons. Lines represent linear regression and 95% CI. *r* denotes Pearson’s correlation coefficient. **(E)** Comparison of firing rates of neurons belonging to different dorsal raphe subnuclei. R, rostral; D, dorsal; V, ventral; I, interfascicular; C, caudal subnucleus; L, lateral wings. Median firing rates and number of recorded neurons per each subnucleus are: R, 2.16 Hz (*n* = 165); D, 2.18 Hz (*n* = 440); V, 2.17 Hz (*n* = 290); I, 1.90 Hz (*n* = 11); C, 2.11 Hz (*n* = 27), L, 2.13 Hz (*n* = 119).

Some recent studies have suggested different electrophysiological properties of DRN serotonergic neurons with respect to their anatomical location. To examine such differences, we reconstructed stereotaxic coordinates of almost all recorded neurons (1052 out of 1087; see “Materials and Methods” Section) and tested whether the firing rate depended on spatial location, as well as compared firing rates of neurons belonging to different subnuclei (Figure 5). Pearson’s correlation revealed no significant difference along the rostrocaudal axis (*r* = 0.012, 95% CI −0.048 to 0.073, *P* = 0.69; Figure 5B) and dorsoventral axis (*r* = 0.009, 95% CI −0.052 to 0.069, *P* = 0.78; Figure 5C), while borderline significance was reached with respect to the lateral position from the midline (*r* = −0.061, 95% CI −0.122 to −0.0001, *P* = 0.046; Figure 5D). In addition, multivariate multiple regression revealed no significant correlation with respect to spatial location (*r* = 0.05, *P* = 0.45). Finally, no differences in firing rate were found among serotonergic neurons belonging to different dorsal raphe subnuclei (*F*_(5,1046)_ = 0.56, *P* = 0.73, ANOVA; Figure 5E). Together, these findings suggest a spatially homogeneous population of serotonergic neurons throughout the DRN.

**Figure 6:**
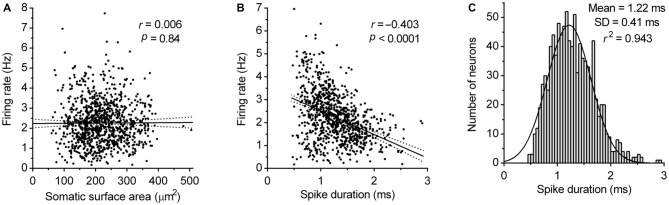
**Correlation of firing rate with neuron size and spike duration. (A)** Correlation between the firing rate and somatic surface area. Symbols represent individual neurons. Lines represent linear regression and 95% CI. *r* denotes Pearson’s correlation coefficient. **(B)** Correlation between the firing rate and spike duration. Symbols represent individual neurons. Lines represent linear regression and 95% CI. *r* denotes Pearson’s correlation coefficient. **(C)** Spike duration histogram of DRN serotonergic neuron population. Curve represents best fit by single Gaussian function.

Next, we examined the dependence of the firing rate on the size of neuron and spike duration. Analysis revealed no correlation between the somatic surface area and the firing rate (Pearson *r* = 0.006, *P* = 0.84; Figure 6A) and moderate negative correlation between the spike duration and firing rate (Pearson *r* = −0.403, 95% CI −0.455 to −0.348, *r*^2^ = 0.162, *P* < 0.0001; Figure 6B). These findings indicate that pacemaker properties of serotonergic neurons do not significantly depend on neuron size and based on *r*^2^ value, that only ~16% of the variability in the firing rate can be explained by variation of spike duration. This suggests that variability in the expression and activity of voltage-gated ion channels determining spike duration, contributes only a small part to pacemaker properties of serotonergic neurons. Finally, the probability density function of spike duration of recorded neurons was found to follow a normal distribution (*r*^2^ = 0.94; Figure 6C), further supporting the hypothesis that DRN serotonergic neurons constitute an electrophysiologically homogenous cell population.

### Spiking Pattern

**Figure 7:**
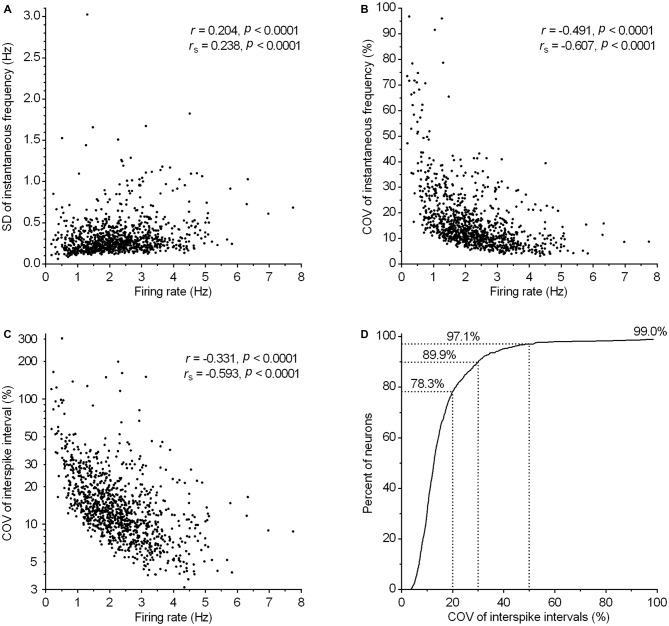
**Regularity of firing in serotonergic neuron population correlates with the firing rate. (A–C)** Graphs show the correlation between various measures of firing regularity and the firing rate. Symbols represent individual neurons. *r* and *r_s_* denote Pearson’s and Spearman’s correlation coefficients, respectively. **(A)** Correlation between SD of instantaneous frequency and the firing rate. **(B)** Correlation between COV of instantaneous frequency (SD of instantaneous frequency/mean instantaneous frequency) and the firing rate. **(C)** Correlation between COV of ISI (SD of ISI/mean ISI) and the firing rate. **(D)** Cumulative frequency distribution of COV of ISI for all recorded neurons. The curve reaches only ~99% because eleven neurons (~1%) having COV of ISI in the range between 116 and 304% are out of scale (for clarity).

Clock-like regular spiking is considered one of the defining electrophysiological properties of serotonergic neurons. Consistently, the majority of recorded neurons exhibited a regular spiking pattern irrespective of firing rate (e.g., see Figure 4D). In order to define quantitatively the spiking regularity of serotonergic neurons, we first wanted to find a measure of regularity which is independent of the firing rate. For that, we run a correlation analysis of potential regularity measures, SD and COV of instantaneous frequency and ISI, vs. the firing rate (Figure 7). We found a weak positive correlation between the SD of instantaneous frequency and the firing rate (*r* = 0.20, *P* < 0.0001, Pearson; Figure 7A) and depending on the test used, a weak (*r* = −0.35, *P* < 0.0001, Pearson) or very strong (*r_s_* = −0.853, *P* < 0.0001, Spearman) negative correlation between the SD of ISI and the firing rate (not shown). There was also a moderate negative correlation between COV of instantaneous frequency and the firing rate (*r* = −0.49, *P* < 0.0001, Pearson; Figure 7B) and between COV of ISI and the firing rate (*r* = −0.33, *P* < 0.0001, Pearson; Figure 7C). Similar values were obtained when correlations were run following exclusion of 1% of neurons with lowest and 1% with highest firing rate and/or neurons showing slow oscillations in firing frequency (see below). These findings indicate an increased regularity of spiking in neurons firing at higher rates. As a consequence, there is no simple measure providing an adequate, rate-independent definition for firing regularity of serotonergic neurons. Importantly, ISI COV, the most commonly used measure of spike train irregularity, is of limited usefulness and although assumed to be rate-independent, shows a stronger correlation with the firing rate than SD of instantaneous frequency.

**Figure 8:**
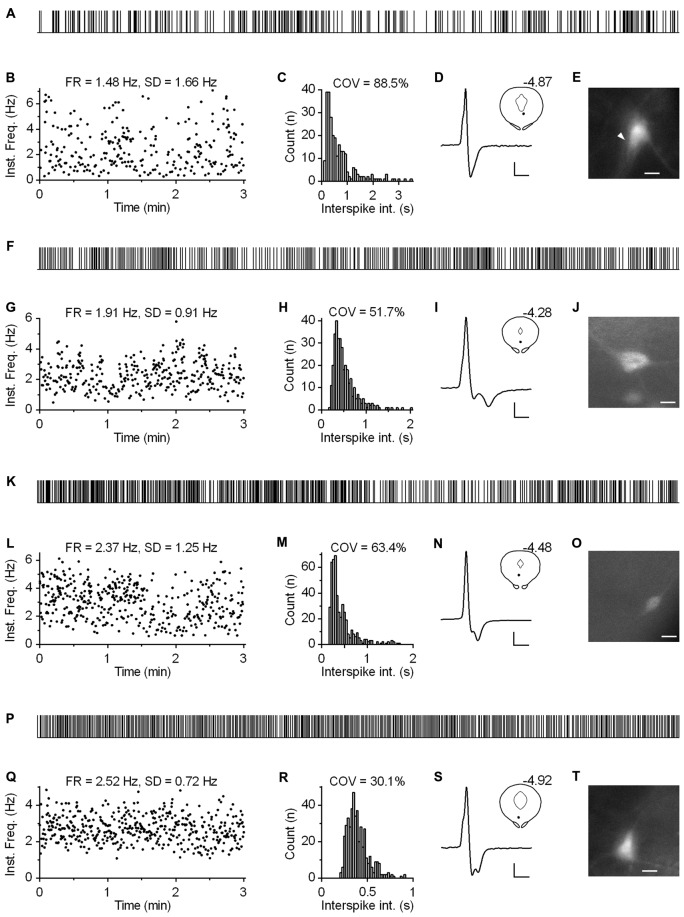
**Subset of serotonergic neurons exhibits somewhat irregular spiking. (A)** 3-min-long spike train of an irregularly spiking serotonergic neuron. TSC mouse, PE-induced firing. **(B)** Time-course of instantaneous frequency of spike train shown in **(A)**. FR stands for the firing rate. SD stands for standard deviation of instantaneous frequency. **(C)** ISI histogram of the train shown in **(A)**. COV denotes variation coefficient of ISI. **(D)** Average spike of the same recording. Scale bars: 8 pA, 1 ms. Inset shows anatomical location of the neuron. Number indicates distance from bregma. **(E)** Fluorescence image of the recorded neuron. Scale bar: 10 μm. **(F–J)** Irregularly spiking serotonergic neuron in PRY mouse. NA-induced firing. **(F)** 3-min-long spike train. **(I)** Scale bars: 15 pA, 1 ms. Inset shows the anatomical location of the neuron. The number indicates distance from bregma. **(J)** Fluorescence image of the recorded neuron. Scale bar: 10 μm. **(K–O)** Irregularly spiking serotonergic neuron in PRY mouse. PE-induced firing. **(K)** 3-min-long spike train. **(N)** Scale bars: 15 pA, 1 ms. Inset shows anatomical location of the neuron. **(O)** Fluorescence image of the recorded neuron. Scale bar: 10 μm. **(P–T)** Moderately regularly spiking serotonergic neuron in PRY mouse. PE-induced firing. **(P)** 3-min-long spike train. **(S)** Scale bars: 15 pA, 1 ms. Inset shows the anatomical location of the neuron. The number indicates distance from bregma. **(T)** Fluorescence image of the recorded neuron. Scale bar: 10 μm.

Regardless of the measure used to assess regularity, it was evident that not all neurons exhibited canonic clock-like spiking. For instance, if the ISI COV of less than 30% and more than 50% are used as a cutoff to classify highly regular and irregular neurons, respectively, then ~7% of recorded neurons could be considered as moderately regular and ~3% as irregular (Figure 7D). Not considering very slow-firing neurons (<0.6 Hz; 28 excluded), most of which would result as irregular according to ISI COV, but not according to instantaneous frequency SD, 1.4% of serotonergic neurons could still be considered as irregular. Most of moderately regular and irregular spiking neurons discharged at a relatively stable rate, but with higher variability in instantaneous frequency and wide positively skewed distribution of ISI. Representative examples of such neurons with firing rates in a range typical for serotonergic neurons (1.5–2.5 Hz) are shown in Figure 8. Irregular as well as moderately regular spiking neurons were located throughout the DRN, and were observed in slices obtained from all three transgenic lines and in both NA- and PE-containing ACSF. Irregular spiking persisted after re-patching the same neuron with a new pipette making it unlikely that it was caused by an interference of the pipette with the recorded neuron. Not different from typical serotonergic neurons, irregular spiking neurons stopped firing in response to a 5-HT1A receptor agonist (30 nM R(+)-8-hydroxy-2-(di-n-propylamino)tetralin, *n* = 3, not shown) and continued to discharge irregularly upon washout. Finally, moderately irregular-type firing was also observed in whole cell recordings in which firing was induced by constant current injection or by PE application (not shown). All together, moderately regular and irregular spiking neurons, apart from higher variability in instantaneous frequency, had properties indistinguishable from those of canonic firing serotonergic neurons.

**Figure 9:**
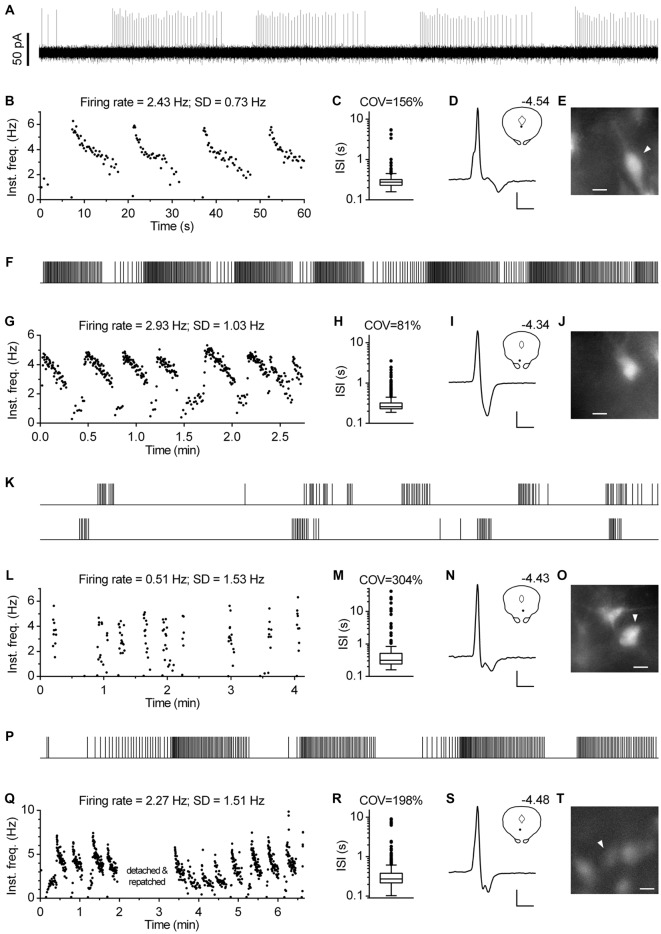
**Subset of serotonergic neurons exhibits low frequency oscillation in the firing rate. (A)** 1-min-long segment of representative recording of serotonergic neuron exhibiting oscillations in firing rate. PCG mouse, PE-induced firing. **(B)** Time-course of instantaneous frequency of the recording shown in **(A)**. SD stands for standard deviation of instantaneous frequency. **(C)** Box plot shows distribution of ISI of the same recording. Log scale. Boxes represent medians and the IQR. Whiskers denote 1.5 IQR. COV denotes coefficient of variation of ISI. **(D)** Average spike of the same recording. Scale bars: 15 pA, 1 ms. Inset shows the anatomical location of the neuron. The number indicates distance from bregma. **(E)** Fluorescence image of the recorded neuron. Scale bar: 10 μm. **(F–J)** Representative recording of low-frequency oscillation in a serotonergic neuron of PRY mouse. NA-induced firing. **(F)** Spike train. Duration, 164 s. **(G)** Time-course of instantaneous frequency. **(H)** Distribution of ISI. Log scale. **(I)** Average spike. Scale bars: 20 pA and 1 ms. Inset shows the anatomical location of the neuron. The number indicates distance from bregma. **(J)** Fluorescence image of the recorded neuron. Scale bar: 10 μm. **(K–O)** Representative recording of serotonergic recording exhibiting intermittent firing with silent periods of up to 40 s. PRY mouse, NA-induced firing. **(K)** Spike train. The bottom part is a continuation of the upper one. Overall duration is 250 s. **(L)** Time-course of instantaneous frequency. **(M)** Distribution of ISI. Log scale. **(N)** Average spike. Scale bars: 15 pA and 1 ms. Inset shows the anatomical location of the neuron. The number indicates distance from bregma. **(O)** Fluorescence image of the recorded neuron. Scale bar: 10 μm. **(P–T)** Low-frequency oscillations persist following repatching of a neuron. PRY mouse, PE-induced firing. **(P)** Spike train of initial 117 s. **(Q)** Time-course of instantaneous frequency. **(R)** Distribution of ISI. Log scale. **(S)** Average spike. Scale bars: 12 pA, 1 ms. Inset shows the anatomical location of the neuron. The number indicates distance from bregma. **(T)** Fluorescence image of the recorded neuron. Scale bar: 10 μm.

**Figure 10:**
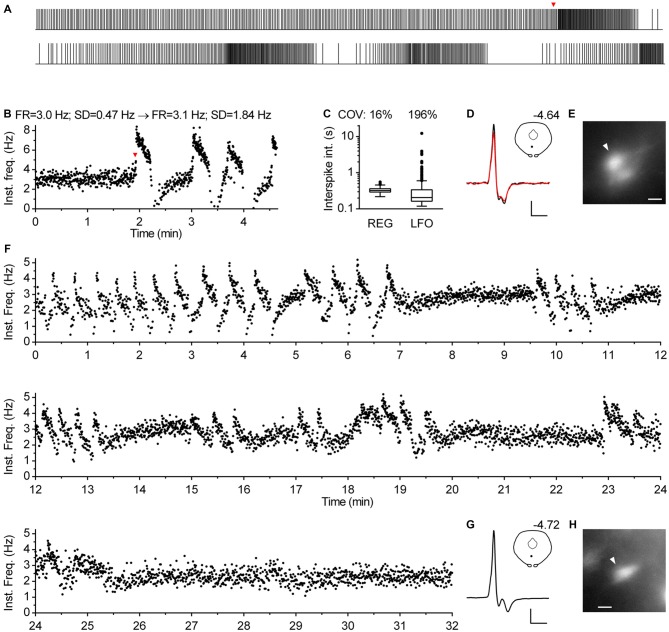
**Serotonergic neurons can spontaneously change spiking pattern. (A–E)** Spontaneous transition from regular to oscillatory firing of a serotonergic neuron in TSC mouse. NA-induced firing. **(A)** Spike train. The bottom part is a continuation of the upper one. Overall duration is 278 s. Firing pattern changed from regular to oscillatory at 115 s (indicated by red arrowhead). **(B)** Time-course of instantaneous frequency of the train shown in **(A)**. Firing rate (FR) was similar during regular firing (0–115 s) and over the course of three full cycles of low-frequency oscillations (115–273 s), while standard deviation of instantaneous frequency (SD) notably increased. **(C)** Box plot shows distribution of ISI during regular firing segment (REG, 0–115 s) and during the segment of three cycles of low-frequency oscillations (LFO, 115–273 s). Log scale. Boxes represent median and the IQR. Whiskers denote 1.5 IQR. COV denotes coefficient of variation of ISI. **(D)** Superimposed average traces of spikes recorded during regular (black line) and oscillatory firing (red line). Scale bars: 15 pA, 1 ms. Inset shows the anatomical location of the neuron. The number indicates distance from bregma. **(E)** Fluorescence image of the recorded neuron. Scale bar: 10 μm. **(F–H)** Prolonged recording from a serotonergic neuron exhibiting LFO. PRY mouse, PE-induced firing. **(F)** Time-course of instantaneous frequency shows multiple transitions in firing pattern. **(G)** Average spike. Scale bars: 15 pA, 1 ms. Inset shows the anatomical location of the neuron. The number indicates distance from bregma. **(H)** Fluorescence image of the recorded neuron. Scale bar: 10 μm.

In approximately one percent of cases (*n* = 12), serotonergic neurons exhibited a particular spiking pattern, characterized by low frequency oscillations (LFO) in firing rate with variable amplitude and the period of oscillation ranging from 10 s to almost a minute. Representative recordings covering the range of observed slow oscillatory behavior are shown in Figure 9. Compared with typical regular spiking serotonergic neurons, those exhibiting LFO had a similar firing rate (~0.5–3 Hz), while their firing regularity was several-fold higher when assessed by SD of instantaneous frequency (~0.7–1.5 Hz) and ~10-fold higher when assessed by COV of ISI (~80–300%). In extreme cases spiking was intermittent, with silent periods lasting up to 40 s (*n* = 3; e.g., Figures 9K–O). LFO-type neurons were observed in slices obtained from all three mouse lines and in both NA- and PE-containing ACSF. There were no obvious differences between regular spiking and LFO-type neurons with respect to size and shape of neurons, spike duration and anatomical location. LFO was also observed following repatching the same neuron (e.g., Figure 9Q), suggesting that it was not caused by pipette interference. In most cases in which LFO was detected, neurons continuously fired with an LFO pattern from the beginning of recording. As we often failed to recognize LFO during the recording, data were acquired for only 2–5 min, a period too short to examine the periodicity of LFO in greater detail. Nevertheless, spontaneous changes in firing pattern were occasionally detected in short recordings (*n* = 2). One such example, where spiking switched from regular to LFO mode is shown in Figures 10A–E. Finally, in one case where LFO was detected online, prolonged recording revealed multiple transitions between oscillatory and regular firing (Figures 10F–H) suggesting that neurons exhibiting LFO are not a separate subpopulation and that LFO is an alternative firing mode of serotonergic neurons.

## Discussion

The loose-seal cell-attached method, which allows recording of intact non-dialyzed neurons, was used to examine spiking activity in a large number of genetically identified DRN serotonergic neurons. This non-invasive recording method and a large sample size permitted us to characterize the spiking properties, which would have likely remained undetected by typical analysis of a lower sample size. The main conclusions of our study can be summarized as follows: (i) in terms of their spiking properties, serotonergic neurons in the DRN represent a homogeneous cellular population; (ii) their regularity of spiking is proportional to the rate of spiking; and (iii) in addition to regular spiking, serotonergic neurons in the DRN can exhibit LFO in firing rate.

In awake state, noradrenergic input exerts a maximal effect on serotonergic neuron firing (Levine and Jacobs, 1992) via activation of α1 receptors (Baraban and Aghajanian, 1980). In brain slice preparations, the noradrenergic input is severed off, but noradrenergic drive may be reinstated by pharmacological activation of α1 receptors (Vandermaelen and Aghajanian, 1983), such as that used in this study. Although α1 agonist-stimulated firing of serotonergic neurons in brain slices mimics an *in* *vivo* situation only to some extent and may be considered as pharmacologically induced rather than intrinsic, it is nevertheless well suited for assessment of electrophysiological properties of individual neurons because it reflects their intrinsic electrophysiological properties relevant for tonic firing in awake state. In this regard, it is noteworthy that firing rate of the DRN serotonergic neurons observed in this study (99% of neurons fired in 0.30–5.81 Hz range) corresponds fairly well with those reported in studies on awake mice. The mean and SD values of serotonergic neuron firing rate found here (2.25 ± 1.06 Hz) are similar to the basal firing rate of optogenetically-identified serotonergic neurons in freely moving mice (1.62 ± 1.70 Hz, *n* = 80; values provided by Li et al., 2016) and are somewhat lower than that of presumed serotonergic neurons during quiet waking (3.21 ± 1.47 Hz, *n* = 194) in head-restrained mice (Sakai, 2011). Furthermore, similar firing rate of the DRN serotonergic neurons (mean = 2.82 Hz) were observed during quiet waking in freely moving cats (Trulson and Jacobs, 1979; Jacobs and Fornal, 1991).

Approximately two thirds of neurons in the DRN are non-serotonergic (Descarries et al., 1982; Jacobs and Azmitia, 1992). Because serotonergic and non-serotonergic neurons are not easily distinguishable based on spike shape and firing properties (Allers and Sharp, 2003; Cohen et al., 2015; and references cited therein), characterization of serotonergic neurons, in particular of those exhibiting atypical spiking patterns, is crucially dependent on the precise identification of recorded neurons as serotonergic. For that, we relied on transgenic mice lines that express fluorescent marker proteins under the control of serotonergic system-specific *Tph2* and *Pet-1* promoters. TPH2 is necessary for serotonin synthesis in the brain and is specifically expressed in the serotonergic neurons of raphe nuclei (Gutknecht et al., 2009). Serotonergic neurons in the raphe can thus be precisely defined on the basis of TPH2 expression and *Tph2* promoter-driven expression of fluorescent reporter genes, such as that of SCFP in the TSC line, which is expected to unmistakably label serotonergic neurons. Pet-1 is an ETS-domain transcription factor whose expression in the brain is restricted to serotonergic neurons (Hendricks et al., 1999; Pfaar et al., 2002). Transgenic mouse lines in which the Cre recombinase expression is driven by the *Pet-1* promoter are well characterized and have been widely used to specifically label serotonergic neurons. There is a possibility, however, that the Pet1-Cre based method does not label all the serotonergic neurons in the DRN (Gaspar and Lillesaar, 2012; Hainer et al., 2015) and it has been shown that Pet1-driven Cre lines have lower specificity and recombination efficiency than Sert-driven Cre lines (Narboux-Nême et al., 2013). In addition, there is evidence suggesting that non-serotonergic neurons could be labeled in Pet1-driven Cre lines. In one Pet1-Cre mouse line it has been shown that Pet1 is expressed also in non-serotonergic neurons in raphe nuclei, with about 1% of Pet-1 expressing neurons being Tph2 negative (non-serotonergic) in the DRN and about 20% in the MRN (Pelosi et al., 2014). Therefore, it cannot be fully ruled out that in Pet1-Cre-based transgenic lines, such as in the PRY and PCG lines used here, some of the fluorescently labeled neurons are non-serotonergic. This possibility seems unlikely, however, because there was a close correspondence between findings obtained using Pet1-Cre based lines and *Tph2* promoter-based TSC line.

Perhaps the main conclusion of this study is that serotonergic neurons in the DRN can be considered as a homogeneous cellular population with respect to their spiking properties. This conclusion is supported by several findings: the probability density function of firing rates follows a normal distribution; multivariate multiple regression shows no correlation between the firing rate and spatial location; there is no difference in firing rate among serotonergic neurons belonging to different dorsal raphe subnuclei; the probability density function of spike durations also follows a normal distribution; and the vast majority of neurons exhibit regular spiking. These findings may seem surprising since there is convincing evidence of functionally distinct serotonergic neuron subtypes in raphe nuclei (Wylie et al., 2010; Calizo et al., 2011; Gaspar and Lillesaar, 2012; Brust et al., 2014; Okaty et al., 2015). Serotonergic neuron diversity is at least in part due to the differences in their developmental history, as different subgroups of serotonergic neurons in raphe nuclei derive from distinct rhombomeric sublineages (Jensen et al., 2008; Wylie et al., 2010; Okaty et al., 2015). Although the heterogeneity of DRN serotonergic neurons cannot be easily explained by diverse cellular origin, as the DRN derives *in toto* from rhombomere 1 (Jensen et al., 2008), recent evidence suggests the existence of distinct serotonergic neuron subtypes also in the DRN (Fernandez et al., 2016). In addition, it has been shown that afferent innervations of the DRN varies along the rostrocaudal axis (Commons, 2009; Soiza-Reilly and Commons, 2011) and that the caudal third of the DRN has afferent innervation more similar to the median raphe nucleus than to the rostral two-thirds of the DRN (Commons, 2015). Furthermore, it was found that a subset of serotonergic neurons do not express 5-HT1A autoreceptors (Kiyasova et al., 2013). Differences were found between serotonergic neurons in the ventromedian subnucleus and lateral wings with respect to electrophysiological properties (Crawford et al., 2010), connectivity and morphology (Crawford et al., 2011), and the expression of G-protein coupled receptors (Spaethling et al., 2014). In contrast to these studies which showed the heterogeneity of DRN serotonergic neurons at multiple levels, but consistent with their common developmental origin, our findings suggest that serotonergic neurons in the DRN represent a homogeneous cellular population with respect to their intrinsic spiking properties. In particular, the α1 receptor-driven firing activity of the DRN serotonergic neurons, which is physiologically important since it is one of the key parameters in determining the brain serotonergic tone, is considerably uniform in spite of the heterogeneity of individual neurons.

Consistent with previous studies, the majority of serotonergic neurons in the DRN exhibited moderately to highly regular spiking. Despite the fact that a quantitative description of spiking regularity was hindered by correlation of regularity measures with the firing rate, it can be concluded that the regularity of spiking of serotonergic neurons is to some extent proportional to their firing rate. This finding is not surprising as, in general, random fluctuations in membrane conductances are expected to introduce more irregularity during a longer-lasting depolarization phase of pacemaking cycle in slower spiking neurons. An additional finding, which was not the main objective of the study design, is that a small fraction of serotonergic neurons exhibit non-canonic firing patterns. Two different modes of atypical firing were observed: ~1% of neurons discharged spikes with relatively high variability in instantaneous frequency (and ISI) while maintaining a fairly stable firing rate; and (an additional) ~1% of neurons exhibited LFO in firing rate. Both atypical firing modes were observed in neurons which were otherwise indistinguishable from canonic-firing neurons and were observed in all three transgenic mouse lines, thus making it highly unlikely that these were “false positive fluorescently labeled” non-serotonergic neurons. In addition, as “high variability” firing can be considered just as an extreme of normal regular firing and since transitions between LFO mode and regular firing were observed, it seems reasonable to conclude that neurons which exhibited atypical firing modes do not represent a separate subpopulation of serotonergic neurons.

Because LFO in firing rate was observed in quite a small percentage of DRN serotonergic neurons, we were surprised to find out that the same phenomenon had been previously observed. In a very first electrophysiological study of rat DRN neurons in brain slice preparations, Mosko and Jacobs (1976) reported that a subset of putative serotonergic neurons exhibits slow oscillation in firing rate. Moreover, the same authors observed the same firing pattern in recordings from chloral hydrate-anesthetized rats (Mosko and Jacobs, 1974). Interestingly, they found neurons exhibiting LFO specifically in the DRN, and not in the median raphe nucleus, and observed this type of spiking persisting for over 1 h. Although the identity of recorded neurons was unknown in these studies, on the basis of their morphology and anatomical location, as well as on the close resemblance of their spiking properties to our findings, at least some seem to be serotonergic. To the best of our knowledge, except for the pioneering studies by Mosko and Jacobs (1974, 1976); there seem to be no other studies reporting LFO-type serotonergic neurons in the DRN. The reason for this may lie in the fact that serotonergic neurons have been commonly identified on the basis of firing regularity and LFO-type neurons were considered irregular, especially if ISI COV was used as a regularity measure. Therefore, it seems likely that over the last 40 years the DRN serotonergic neurons exhibiting LFO spiking mode have been misidentified as non-serotonergic.

LFO is observed in a very small percentage of neurons. The proportion of neurons exhibiting LFO may result higher in recordings of longer duration. Although our recordings are too short for periodicity analysis, it seems that at least some of the moderately regular and irregular spiking neurons exhibited LFO-like spiking pattern. The fact that LFO-type neurons have been rarely observed does not preclude the possibility that *in vivo* a higher fraction, or even all serotonergic neurons in the DRN can discharge in LFO mode. In that respect an analogy can be drawn with spike doublets firing mode, which has not been observed *in vitro*, but has been observed in recordings from serotonergic neurons in anesthetized rats and mice (Hajós et al., 1995, 2007; Montalbano et al., 2015). The functional implications of LFO spiking mode are currently unclear. Further *in vivo* studies are needed to elucidate the relationship between LFO spiking mode and sleep/wake/arousal states as well as particular behaviors. At present, it can only be concluded that in terms of their intrinsic spiking properties, serotonergic neurons in the DRN are homogeneous and that, at least a subset of them, can discharge in LFO mode.

## Author Contributions

BM designed the study, analyzed data, and wrote the manuscript. AM performed and analyzed experiments. LP and CG designed and produced *TSC* mouse line. RC designed and coordinated the study.

## Funding

This work was supported by grants from the University of Florence and Ente Cassa di Risparmio di Firenze (ECRF-2007-0758). AM was recipient of a fellowship from the Regione Toscana and Aziende Chimiche Riunite Angelini Francesco A.C.R.A.F. SpA (POR CRO FSE 2007-2013: 5-HT@DRUGeMOOD).

## Conflict of Interest Statement

The authors declare that the research was conducted in the absence of any commercial or financial relationships that could be construed as a potential conflict of interest.

## Footnotes

^1^https://imagej.nih.gov/ij/
